# Iconic Gestures for Robot Avatars, Recognition and Integration with Speech

**DOI:** 10.3389/fpsyg.2016.00183

**Published:** 2016-02-17

**Authors:** Paul Bremner, Ute Leonards

**Affiliations:** ^1^Bristol Robotics Laboratory, University of The West of EnglandBristol, UK; ^2^School of Experimental Psychology, University of BristolBristol, UK

**Keywords:** human-robot interaction, gestures, humanoid robotics, tele-operated robot, multi-modal communication

## Abstract

Co-verbal gestures are an important part of human communication, improving its efficiency and efficacy for information conveyance. One possible means by which such multi-modal communication might be realized remotely is through the use of a tele-operated humanoid robot avatar. Such avatars have been previously shown to enhance social presence and operator salience. We present a motion tracking based tele-operation system for the NAO robot platform that allows direct transmission of speech and gestures produced by the operator. To assess the capabilities of this system for transmitting multi-modal communication, we have conducted a user study that investigated if robot-produced iconic gestures are comprehensible, and are integrated with speech. Robot performed gesture outcomes were compared directly to those for gestures produced by a human actor, using a within participant experimental design. We show that iconic gestures produced by a tele-operated robot are understood by participants when presented alone, almost as well as when produced by a human. More importantly, we show that gestures are integrated with speech when presented as part of a multi-modal communication equally well for human and robot performances.

## 1. Introduction

Based on the idea that embodiment leads to stronger social engagement than a screen (Adalgeirsson and Breazeal, 2010; Hossen Mamode et al., 2013), we wondered whether a viable alternative for telecommunication is to use a tele-operated humanoid robot as an embodied avatar in a remote location. In previous work with robot avatars they have been shown to improve social presence of a remote operator (Tanaka et al., 2015), and their salience to people in the robot's presence (Hossen Mamode et al., 2013), relative to more traditional telecommunication media (audio and video).

In order for a robot avatar to be a viable communication method it must be capable of transmitting human interactive behavior. In everyday communication people can be observed performing arm gestures alongside their verbal communications (McNeill, 1992; Kendon, 2004). Though there is much debate on whether such gestures have a communicative value for observers, a recent meta-analysis of the literature concluded that they are of communicative value (Hostetter, 2011). Indeed, a number of studies in the human communication literature demonstrate observers of co-verbal gestures comprehend information from them (Cassell et al., 1999; Kelly et al., 1999; Beattie and Shovelton, 2005, 2011; Cocks et al., 2011; Wang and Chu, 2013). Hence, we are motivated to investigate the use of gesturing on a humanoid robot avatar to capitalize on the reported benefits (salience and social presence), while still maintaining multi-modal communication efficacy.

To transmit the multi-modal communications of a human operator, we have developed a tele-operation interface that uses motion tracking of the operators arms, and audio streaming, to replicate their communication on a NAO robot (Aldebaran Robotics, Gouaillier et al., 2009). By using this implicit control method we aim to allow an operator to communicate as they would face-to-face. Before being able to investigate the benefits of embodiment over video in telecommunication, and interaction benefits of gestures, we first need to demonstrate the capability of the system to reproduce comprehensible gestures on the robot; thus, this is the first aim of the work presented here.

Which kind of gestures are particularly important in human–human communication, and how they can be shown to add communicative value, underpins our approach to evaluating multi-modal communication on a robot avatar. Within the literature on gestures in human interaction a number of schemes have been proposed to classify them according to their form and function (Ekman, 1976; McNeill, 1992; Kendon, 2004).

Iconic gestures are a key class of gestures from the classification scheme proposed by McNeill (1992). Iconic gestures are those that have a distinct meaning, they are of a form that either reiterates or supplements information in the speech they accompany. They typically convey information that is more efficiently and effectively conveyed in gesture than in speech, such as spatial relationships and motion of referents (Beattie and Shovelton, 2005), or the way in which an action is performed (termed manner gestures) (Kelly et al., 1999). Hence, multi-modal communication can be said to be more effective and efficient at conveying information between speaker and listener than uni-modal communication, i.e., taking less time to convey the desired message, and in a clearer way (Beattie and Shovelton, 2005). Given the high communicative value of iconic gestures, here we investigate their use in robot avatar communication.

For human-human communication, a number of approaches have been taken to establish the communicative value of iconic gestures, by examining whether the information understood by observers of multi-modal communication differs from uni-modal communication. One suggested value of gestures is that they improve how memorable the speech they accompany is. Hence, participants' ability to recall details of speech delivered with and without different gestures has been tested (e.g., Cassell et al., 1999; Kelly et al., 1999). Analysis of results for such experiments is non-trivial, and depends strongly on how easy the stimulus material content is to remember.

An alternative approach was suggested by Beattie and Shovelton (2005), whereby participants were asked questions about short multi-modal vignettes, the answers to some of which were only contained in the gestural channel. However, in such an approach it might be difficult to distinguish between speech and gesture integration, and contextual inferences (Beattie and Shovelton, 2011).

To avoid confounds such as the ones potentially inherent in the approaches described above, we decided to base our experiments on a seminal study presented by Cocks et al. (2011). We adapted their design for use with the NAO robot and our tele-presence control scheme (see Section 2). In their study, participants were presented with a series of actions conveyed either through speech alone, gesture alone, or an iconic (manner) gesture accompanying speech, and asked to select, from a set of images of actions one that best matches what was communicated. The authors were able to clearly distinguish and compare understanding of actions both in uni-modal and multi-modal communication. Hence, their method was able to evaluate integration of information from the two communication channels, a process vital for the utility of co-speech iconic gestures (Cocks et al., 2011).

One of the aims of the work presented here is to investigate whether the integration of speech and gesture occurs for a non-human agent, such as a robot, in the same way that it does for a human. Knowledge in this regard is as yet very limited. Speech and gesture integration for robot-performed pointing (deictic) gestures has been investigated (Ono et al., 2003; Cabibihan et al., 2012b; Sauppé and Mutlu, 2014), this showed that relative locations of referents could be better understood by using gestures to supplement speech information. While these studies provide some evidence for speech and deictic gesture integration, iconic gestures have yet to be examined. Moreover, to the best of our knowledge, it has never been investigated whether this integration process is as reliable in robots as it is in people.

A key issue in robot gesturing, is joint coordination and motion timing. Work on how the human brain processes gestures suggests this may be of importance to gesture recognition, and hence in studying speech and gesture integration. In their recent meta-analysis of studies concerning the neural processing of observed arm gestures Yang et al. identified three brain functions associated with gesture processing: mirror neurons, biological motion recognition, and response planning (Yang et al., 2015). Of particular relevance here are mirror neurons, part of the brain associated with performing actions that fire when those actions are recognized. Gazzola et al. showed that mirror neurons still fire when observing some robot motion (Gazzola et al., 2007). However, they suggested that this depends on identification of the goal of the motion. With gesture, the motion goal is often not clear, and so mirror neuron based gesture recognition may instead rely upon identification of motion primitives, component parts of gestural motion based upon muscle synergies in the arm (Bengoetxea et al., 2014).

A potential advantage in our study is we might overcome any scripting-related issues by using our tele-operation control scheme to copy both the shape, timing and joint coordination of human movement. Note, however, that even a tele-operation control system is limited by the design and the degrees of freedom of the robotics system used. Moreover, the non-biological appearance of the robot may interfere with identification of the gestures. Hence, we included testing conditions that allowed us to evaluate the comprehensibility of the gestures produced with our system when presented on their own.

In this paper we aim to address the following research questions: (1) can iconic gestures performed with our tele-operation system be identified?; (2) is performance comparable to when the same gestures are performed by a person?; (3) are iconic gestures performed using our tele-operation system integrated with speech?; and (4) is integration as efficient for robot performed multi-modal communication as human performed multi-modal communication?

In detail, we pre-recorded a set of communications consisting of verb phrases and appropriate iconic gestures produced by the robot using our tele-operation system, and a matching set by a human actor. The same actor was used for producing the robot stimuli and the human stimuli (recorded on video) to make the conditions as closely matched as possible. The recorded stimuli were then used in an experimental study adapted from the human–human communication literature (Cocks et al., 2011) to investigate whether hand gestures on their own were comprehensible for both robot and human, and whether they could be integrated with speech.

To evaluate integration, we established whether the understanding of the observers' was changed as compared to speech or gesture alone. Understanding was also directly compared for the human (on video) and the robot (embodied replay of recorded communications) within the same observers. We sought to establish the extent of integration benefit achievable with robotic communication, relative to the one observed for a human communicator. We used videos of human gestures in our study to ensure identical stimuli for all participants. We reasoned they would be as efficient as live performances, given high recognition and integration rates (close to ceiling) were observed using video stimuli, in the study on which our work is based (Cocks et al., 2011).

An additional motivation for our comparison of human video communication with a physically present robot is that it allows us to evaluate the differences between these two modes of telecommunication for multi-modal communication. If the performance of gesture understanding and integration for the robot avatar is comparable to video communication, it will enable further work on the salience and utility of these gestures in an interactive context. Beyond the application of the results to the utility of the NAO robot as an avatar, the tele-operated approach allows us to make more general inferences for the design of autonomous communicative robots.

Directly comparing participants' comprehension of iconic gestures and their integration with speech for human and robot performers (in a single experiment) allows us to eliminate a range of confounds that make it difficult to compare findings within the literature. To the best of our knowledge we are the first to make this direct comparison.

This paper is an extended version of our work published in Bremner and Leonards (2015a). We extended our previous work by adding in depth analysis of the gestures used, and the performance of the tele-operation system in reproducing these gestures. Additionally there is far more detailed discussion of our results, including implications of related work in neuroscience on human gesture processing.

## 2. Materials and methods

We conducted an experimental study with 22 participants (10 female, 12 male), aged 18–55 (*M* = 34.80 ± 10.88*SD*), all of whom were Native English speakers. Participants gave written informed consent to participate in the study, in line with the revised Declarations of Helsinki (2013), and approved by the Ethics Committee of the Faculty of Science, University of Bristol.

Stimuli consisted of a series of pre-recorded communications, these were either speech alone, gesture alone, or speech and gesture. Each communication was performed by either the human actor (on video) or the NAO robot (physically present). Video was used for the human stimuli to ensure repeatability, and to allow direct comparison of data obtained for speech and gesture integration in dependence of the type of communicator: human or tele-operated robot. Hence, the experiment used a 2 (performer) × 3 (communication mode) within-subjects design.

### 2.1. Tele-operation system

**Figure 1:**
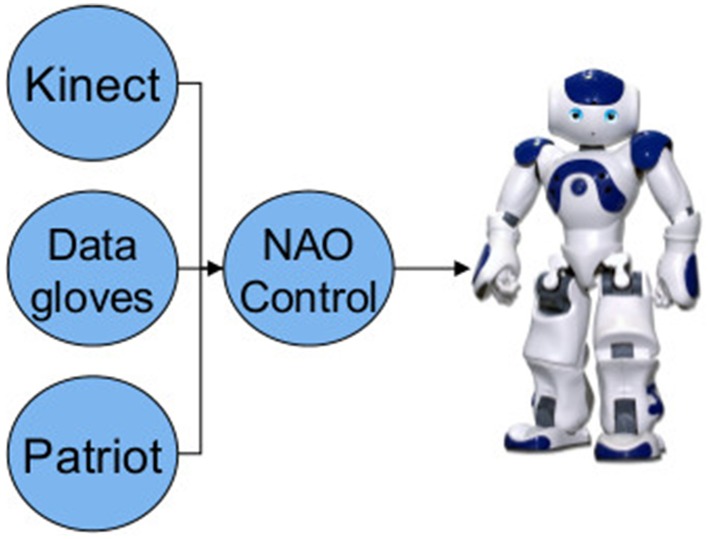
**Control architecture of the tele-operation system**. Circles represent ROS nodes. © 2015 IEEE. Reprinted, with permission, from Bremner and Leonards (2015a).

To reproduce gestures performed by a human actor on the NAO humanoid robot platform from Aldebaran Robotics (see Figure 1, for specifications see Gouaillier et al., 2009), we designed a motion capture based tele-operation system. The system was built using the ROS framework. Architecturally, ROS can be described as a computation graph made up of software modules (termed nodes), communicating with one another over edges (Quigley et al., 2009). Communication is built on a publisher/subscriber model where a node sends a message by publishing it, and nodes using that message subscribe to it.

ROS offers a number of advantages that make it well suited to our system. Firstly, its communication architecture means that the system is inherently modular, so if one node fails the others can keep running while the failed node is restarted. Secondly, this modularity means nodes can be easily modified independently, only needing to adhere to correct message structure, making the system easily extensible. Thirdly, nodes can be written in different programming languages, here some nodes use C++ and some Python. Finally, ROS is well documented with a large library of existing nodes on which to base our work, speeding development time. Hence its use over viable alternatives such as YARP (Metta et al., 2006) or URBI (Baillie et al., 2008).

In our tele-operation system we have developed separate nodes to gather kinematic information of the human tele-operator from several sensor systems. Each sensor node then publishes its data as ROS messages, a NAO control node subscribes to these message streams and then calculates the required commands that are then sent to the robot. Figure 1 shows the system architecture schematic. Audio streaming was handled separately from ROS using the GStreamer media framework to develop a NAO module and corresponding PC application to allow streaming of audio to the robot.

In order to ensure that gestures are reproduced on the robot as closely as possible to the original human motion, hand trajectories, joint coordination and arm link orientations must be maintained. To this end arm link end points (i.e., shoulder, elbow and wrist) are tracked using a Microsoft Kinect sensor; the Nite skeleton tracker API from OpenNI is used to process the Kinect data and produce the needed body points. A Kinect node was written with the Nite API that uses the arm link end points provided by the skeleton tracker to calculate unit vectors for the upper and lower arm in the operator's torso coordinate frame^1^, these were then published as ROS messages. Sensor update rate was 30 Hz.

**Figure 2:**
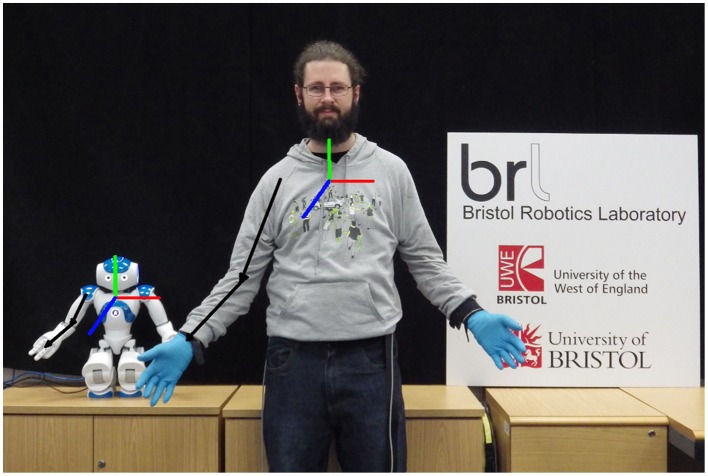
**A tele-operator pose reproduced on the NAO robot**. Black arrows indicate the directions of the unit vectors along the arm links, the coordinate frame of the torso is shown in RGB (XYZ). © 2015 IEEE. Reprinted, with permission, from Bremner and Leonards (2015a).

The arm unit vectors are then used by the NAO control node to calculate robot arm joint values that align the arm links of the robot with those same unit vectors in the torso coordinate frame of the robot^1^. An example mapping between human and robot arm positions is shown in Figure 2. Data from the Kinect were subject to high levels of noise, consequently the joint angles were smoothed using a moving average filter with a 10 frame window.

The filtering process added undesirable delay to the robot commands. Consequently, each filtered value is then modified by adding a trend term, calculated for each joint as a 10 frame moving average of the change in position each frame, then scaled by a factor of 4 (empirically determined) to produce a command similar to, but slightly ahead of, the raw value. To prevent overshoot due to sudden changes in velocity the filtered output was limited to deviate from the un-filtered value by an empirically determined maximum threshold value (0.04 rad). The NAO control module executed these commands to ensure the joints are still in motion when new commands are received, to do this it sent motor demands to execute the motion over a longer period than the update rate would require, so the controller doesn't decelerate more than demanded by the control node. This process utilized the inbuilt NAO position controllers to counteract commands being ahead of the raw value (resulting from the trend term in the filter), and thus allowed smooth handling of the stream of position demands.

Due to limitations of the resolution of the Kinect when viewing the full body, it is not able to provide all degrees of freedom (DoF) required. Specifically, finger flexion and extension, and hand rotation relative to the forearm (pronation/supination). To overcome these limitations additional sensors were used: a Polhemus Patriot provides pronation/supination, and 5DT data gloves provide finger bend information. ROS nodes were developed for each of the additional sensors, which publish that data as ROS messages at 30 Hz. The NAO node processes this additional data to calculate the needed joint angles for the robot. It then combines the calculated angles for all arm joints into a single message to send to the robot each command cycle.

### 2.2. Phrase and gesture selection

In order to evaluate whether the tele-operation system could produce comprehensible gestures, and whether the produced gestures were integrated with speech they accompany, we first had to determine a suitable set of phrases and accompanying gestures. We selected 10 verb phrases, depicting common actions (e.g., I played, I opened), chosen from those used by Cocks et al. (2011), see Table 1 for the full list. An important feature of the phrases selected is that they have more than one manner in which they can be conducted, and these manners can be conveyed with manual gestures.

**Table 1:** **The 10 verb phrases, their preconceived meanings when accompanied with each of the two manner gestures (integration target), description of the manner gestures**.

**Phrase**	**Integration target**	**Gesture description**
I Cleaned	1. Dusting a lamp	One hand open flat, palm down, moves diagonally from center line, at shoulder height, down and outwards toward periphery and then back again twice
	2. Scrubbing a pan	One hand moves in a horizontal circle in center, hand is in a power grip, palm down
I Cut	1. Cutting with a craft knife	One hand moves from center line, horizontally outwards toward periphery, hand in a precision grip
	2. Chopping into a melon	flat vertical palm moves in a downward chopping motion, in periphery
I Fixed	1. Hammering a nail	One hand in a vertical closed power grip moves up and down twice in a curved path, in periphery
	2. Sticking paper with tape	Both hands in precision grip, palm down, hand length apart, move downwards as if pressing something down, in center center
I Lit	1. Pulling a light pull	One hand in a vertical closed power grip moves to shoulder height arm partially extended, then moves vertically downwards, in periphery
	2. Pressing a light switch	One hand, with index finger extended, moves diagonally up and out away from the torso to finish just below shoulder height, in periphery
I Measured	1. Pouring liquid into a measuring jug	One hand adopts an vertical open power grip, the other a vertical precision grip above and to the side of the other hand, the wrist is rotated in a pouring motion, both hands in center center
	2. Using a tape measure	Both hands adopt a precision grip, palm down, and move close together in center center, right hand then moves horizontally away from the stationary left hand, toward periphery
I Opened	1. Pulling open a door	One hand reaches out away from the body, adopts a vertical precision grip then retracts straight backwards, in periphery
	2. Opening a book	one flat hand, horizontal, palm down in center center, hand moves up and out toward periphery with wrist rotation to flip hand over
I Paid	1. Signing a check	one hand in a precision grip tracing a curling path from the center out to the periphery
	2. Handing over cash	One hand open, palm horizontal and face up, hand moves out and up as if presenting an object on the hand, in periphery
I Played	1.Playing chess	One hand adopts a horizontal grip, palm down, in center, near the body then follows an arcing trajectory forwards and releases the grip
	2. Playing a cello	One hand, in a horizontal fist, palm down, moves back and forth across the center-line of the body
I Read	1. Reading a newspaper	Both hands in vertical closed power grip shoulder width apart
	2. Reading a book	Both hands in vertical closed power grip a hand length apart, in centre
I Rubbed	1. Using a pencil eraser	One hand, horizontal closed power grip, palm down, moves left to right rapidly near centreline of body
	2. Rubbing a balloon	One hand partially open power grip moves vertically up and down twice, in periphery

For each phrase two different iconic (manner) gestures were determined that conveyed manner in which the action was performed. This is an extension of the original design as presented by Cocks et al. (2011), who used only a single gesture for each phrase. We made this modification for two main reasons, firstly to give us a larger range of gestures to evaluate for comprehensibility on the NAO robot; secondly, and more importantly, to better evaluate speech and gesture integration. Indeed, we would argue that showing two different shifts in meaning from a speech only interpretation provides stronger evidence for integration.

To select appropriate gestures there are a number of factors that must be considered. The primary aim for the gestures is that they are sufficiently vague that they might convey multiple possible meanings when viewed without words; at the same time, they must still be interpretable without the need for speech. This requirement also served to increase the ecological validity of the gestures being used, as they were close to those that might be performed in everyday speech. Note that this clearly contrasts with a precise pantomime gesture of a particular action, which is likely to have only one interpretation, and which is rarely used in normal conversation (Cocks et al., 2011).

Another important requirement was that the gestures had to be performable by the NAO robot, such that a fair comparison could be made between gestures performed by a person and the robot. While the NAO robot does have degrees of freedom in its arm such that it can cover a wide range of human-like movements (Gouaillier et al., 2009), it does have a number of limitations relevant to the performance of gestures. The most important of these is that the NAO only has three fingered, one degree of freedom hands, where all fingers open and close simultaneously. Hence, NAO is not capable of much in the way of hand-shapes, a key component in many human upper limb gestures. To accommodate for this restriction we selected gestures which mainly comprised arm movements, for which precise hand shape and finger movements were deemed less critical. Note further, the NAO robot also has only one degree of freedom in the wrist (pronation/supination), compared to the 3 degrees of freedom in the wrist of humans, a reduced range of flexion in the elbow, and a safety algorithm to prevent the two hands from colliding. While we have tried to select gestures that are relatively unaffected by these restrictions, in order to maintain ecological validity, the human performer/tele-operator was not instructed to accommodate any of these factors.

The final selection of gestures are described in Table 1. To simplify descriptions, and aid analysis of gesture features, the description of gesture space proposed by McNeil was used (McNeill, 1992). To further aid description we use the terms power grip: gripping with the whole hand, and precision grip: gripping with the finger tips.

### 2.3. Materials and procedure

The experiment stimuli consisted of recordings of the 10 verb phrases detailed in Table 1. Each verb phrase was performed twice, once for each of the iconic (manner) gestures that portrayed how the action was performed. Two stimulus sets were recorded, the human performer stimuli was recorded using a digital video camera, the robot stimuli was recorded using the tele-operation system. In order to avoid inter-individual variability in action performance, the same human actor performed both human and robot stimuli.

To avoid possibly distorting participant perceptions due to the presence of the data-gloves necessary for tele-operation, the two stimulus sets were recorded separately. In order to ensure that the stimulus sets were as similar as possible, prior to performing without the data-gloves the actor reviewed the video of each tele-operation performance. The two recordings of each stimulus item were compared, and, where necessary, repeat performances were recorded.

The robot communication stimuli were created by recording the messages transmitted by the sensor nodes using the built in recording capabilities of ROS. Audio was captured using the GStreamer based software module. To allow immediate verification, the robot was controlled and streamed to during recording.

The human video stimuli and the recorded tele-operation stimuli were then edited to produce a set of presentations lasting approximately 5 s each, in three conditions: verbal only condition (V; audio only no performer movement); gesture only condition (G; gesture visible but audio not played); verbal-gesture condition (VG; gesture seen and verbal phrase heard). In both G and VG conditions, there were two different manner gestures so two presentations were created for each verb phrase. Hence, each action phrase came in five different versions per performer (V, G1, G2, VG1, VG2).

To create the human stimuli the audio recorded during the robot performances was added to the videos of the human performance (i.e., replacing the original audio). Hence, identical audio was used for both robot and human performances in the 3 condition with a verbal component. Audio-information was overridden for the human stimuli to make sure that the audio information provided was identical between both human and robot stimuli. To prevent any lip-syncing issues, and eliminate the possibility of facial gesture effects, the human performer's face was obscured in the video. The relative timing of speech and gesture for the robot performances was based on video recorded of the robot captured during stimulus recording with the tele-operation system.

There were 10 experimental conditions in total: five communication modes (V, G1, G2, VG1, VG2) for each of the two performers. Ten action phrases were used in each experimental condition; hence, each participant responded to 100 different trials. The trials were split into 10 blocks, each containing all 10 phrases, and all 10 experimental conditions. To prevent ordering effects, trial presentation order was counterbalanced across and within blocks by means of pseudo-randomization using partial Latin squares.

Following each stimulus presentation, participants were presented with a set of six color photos of people performing actions on the (12.1 inch) screen of a response laptop, and were asked to select one. To do so they clicked with the laptop's mouse cursor on the photo they thought most closely matched what had been communicated; doing so moves on to the next stimulus presentation. The layout of the images, and hence the location of the target(s) on the response screen, were randomized between conditions and between phrases. Presentation of the response images, and recording of responses was done using the PsychoPy software (Peirce, 2007). Average experiment time was 20 min.

**Figure 3:**
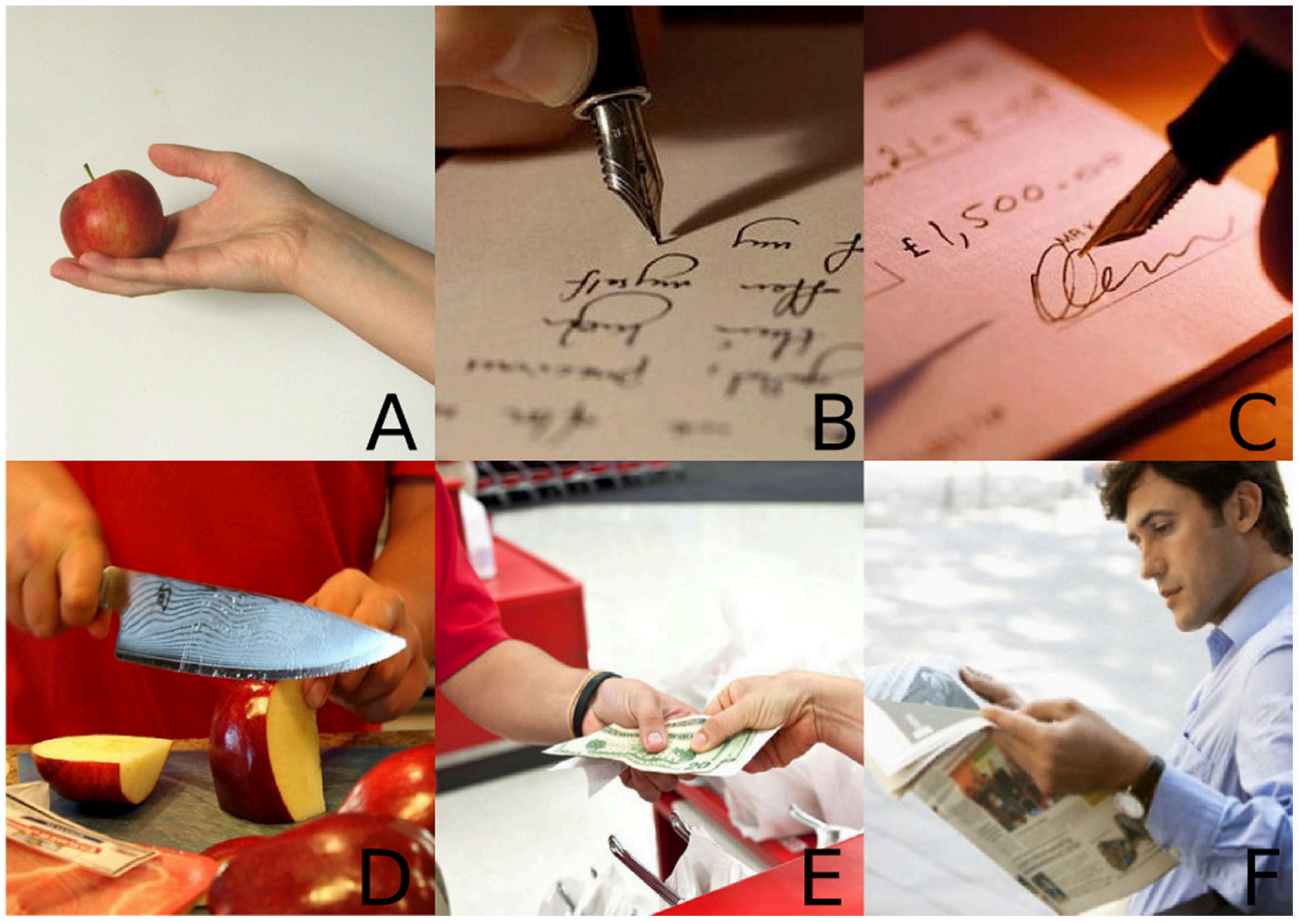
**The response images for “I paid”: (A,B) match only the gestures; (C,E) are the integration targets, both of which match the speech only condition; (D,F) are the unrelated foils**. © 2015 IEEE. Reprinted, with permission, from Bremner and Leonards (2015a).

The response image set for each phrase consisted of: a gesture only target for each gesture, that matched the corresponding gesture but not the speech; an integration target for each of the two manner gestures, which matched the corresponding speech and gesture combination; a pair of unrelated foils, not matching either the gesture or the speech, each one linked semantically to one of the gesture-only images (Figure 3 shows an example set, for “I paid”). For a particular gesture, one gesture only image and one integration target were both semantically congruent with it, so should have been selected with equal likelihood in the G condition. Both of the integration targets were semantically congruent with the speech, so in the V condition each should have been selected with equal likelihood. In each of the VG conditions only a single integration target was congruent for that particular speech and gesture combination, hence it should be the most probable image selection.

**Figure 4:**
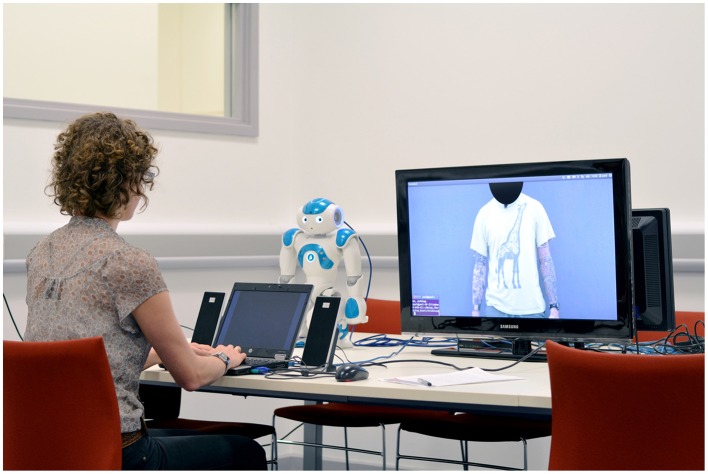
**Set-up for the experiment**. © 2015 IEEE. Reprinted, with permission, from Bremner and Leonards (2015a).

Figure 4 shows the experimental set-up. The video screen and the NAO robot were both positioned 57 cm from the participant. A 32 inch wide-screen TV was used to display the video stimuli, thus, the human performer and robot appeared to be of a similar size. The start of each trial was signaled to the participant by playing a tone and displaying either human or robot on the response laptop for 1 s to indicate which presenter was next. This allowed the participant to concentrate on the correct presenter from the outset of each trial. Each trial consisted of playback of the performance of the phrase, followed by automatic display of the response image set. Each trial was initiated by the experimenter after the participant had completed the previous trial; the experimenter was sat out of view of the participant. Prior to the experimental trials, participants performed two practice trials to ensure they understood the experimental procedure.

## 3. Results

### 3.1. Gesture comprehension

**Figure 5:**
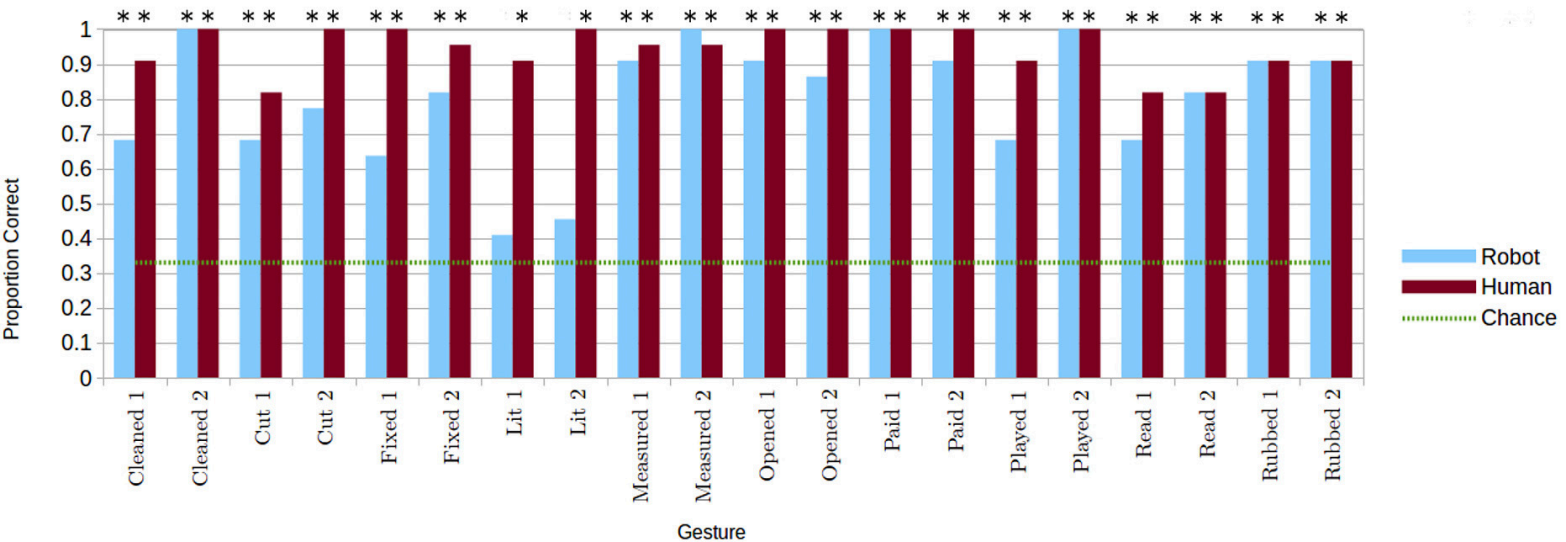
Proportion of correct identifications of each gesture for the two performance conditions, when gestures are presented alone. Correct gesture identifications significantly greater than chance indicated with ^*^p < 0.05. © 2015 IEEE. Reprinted, with permission, from Bremner and Leonards (2015a).

Gesture comprehension was tested by calculating the proportion of correct responses in the conditions with only gestures. To evaluate each gesture, in both performance conditions, a chi-squared test was used to compare the proportion of correct responses for that gesture with chance (of the six images in the response set two were the correct answer, so chance was at 0.33). These results are shown in Figure 5. Almost every gesture (with the exception of both the “I lit”gestures in the robot condition) was identified significantly better than chance in both human and robot conditions, with high average proportions of correct responses (*M*_*human*_ = 0.943 ± 0.065*SD*; *M*_*robot*_ = 0.802 ± 0.17*SD*). A Wilcoxon signed rank test (used as the data did not meet assumptions needed for a parametric test) revealed a significant difference between performers (*p* < 0.001) for the same gestures even excluding the “I lit”gestures.

It is apparent from Figure 5 that sizeable differences in gesture comprehension between performers existed only for some of the gestures examined. Hence, the data were further analyzed, on a per gesture basis, to find for which individual gestures there were significant differences in recognition rate between performers. As the data is binomial and paired (each participant viewed human and robot performances of each gesture), we used an exact McNemar test to evaluate differences. An exact McNemar test for each gesture revealed gestures were identified correctly significantly more frequently in the human performances than in the robot performances for lit1 (*p* = 0.00098), lit2 (*p* = 0.00049), and fixed1 (*p* = 0.00781). Cut2 approached being significantly more frequently correctly identified in human performances than in in robot performances (*p* = 0.0625). There were no other significant differences in gesture identification between human and robot performance conditions. Note, however, that these results^2^ need to be treated with caution as performance was almost at ceiling, resulting in small values for the dichotomous variables used in the test calculations.

**Figure 6:**
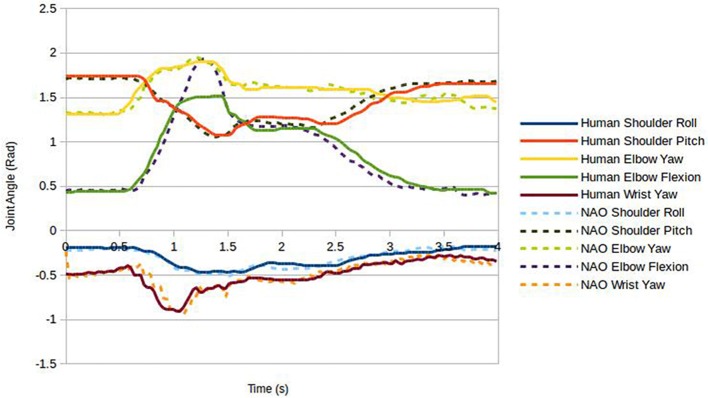
**Joint values during the Lit1 gesture for human and robot performers**.

**Figure 7:**
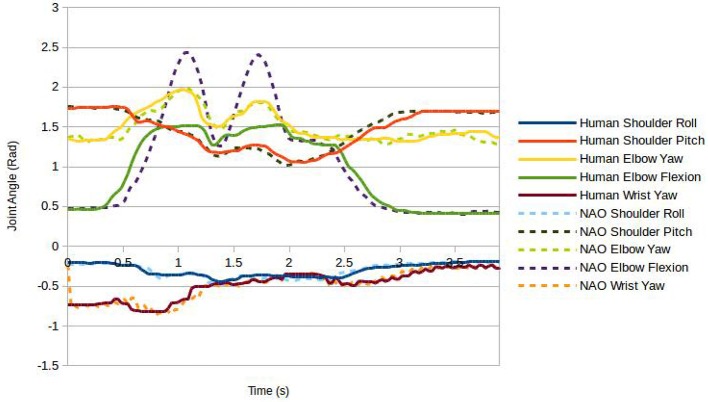
**Joint values during the Fixed1 gesture for human and robot performers**.

In order to investigate possible sources for the difference in gesture comprehension found between human performer and robot, controller performance was further analyzed for two of the gestures; namely those for which significant differences had been reported—lit1 and fixed1. First we compared the physical movement profiles: for this, the recorded robot joint values over the duration of each gesture were plotted along with the joint values for the human performer as recorded by the Kinect (Figure 6, Lit1, Figure 7, Fixed1). It is clear from the graphs that joint co-ordination and velocity profiles, and hence hand trajectories, are very comparable between human and robot for the two gestures analyzed. However, two common differences can be observed in both plots, firstly the elbow flexion has a limited range of motion on the robot relative to the human, decreasing the amplitude of the peak of the gesture (approximately 15% reduction in vertical travel); further, they have a very brief pause at the top of the stroke.

Secondly, the predictive filter caused the robot joints to accelerate at a slightly different rate to the human joints when the human joint velocity was at certain values; this resulted in those joints finishing their motion approximately 0.1s early. It is hard to quantify the significance of these differences. Although they appear relatively small, critical visual examination of the robot motion on these two gestures may provide further insight. In both cases the hand trajectory is largely as expected and joint coordination appears on visual inspection human-like. However, the slightly shorter vertical travel is noticeably different from what is expected for these two actions, but vertical travel is still clearly perceptible. Further, in the human version of these gestures ulnar/radial deviation in the wrist is used, a degree of freedom lacking in the NAO robot. A pause in the gesture is barely perceptible, and only in the oscillatory motion in fixed1, appearing less smooth than expected.

To provide further insight into differences in gesture performances, the gestures lit2, cut2, played1, and cleaned1 were also analyzed by visual inspection. Though not significantly different in identification between performers, cut2, played1 and cleaned1 all led to differences in identification performance between human and robot performer (5). Similarly to lit1 and fixed1, cut2 and played1 showed reduced vertical travel for the robot performance due to a reliance on elbow flexion. It is also apparent from lit1, lit2, cut2, and cleaned1 that the wrist rotation sensor did not always give accurate readings. As a result, wrist orientation differed visibly from the human version of these gestures. Although we would have thought that hand-shape itself should play only a minor role in these gestures, in lit2, and cut2, a fairly particular hand-shape was adopted by the human which the NAO was unable to approximate well enough.

### 3.2. Speech and gesture integration

**Figure 8:**
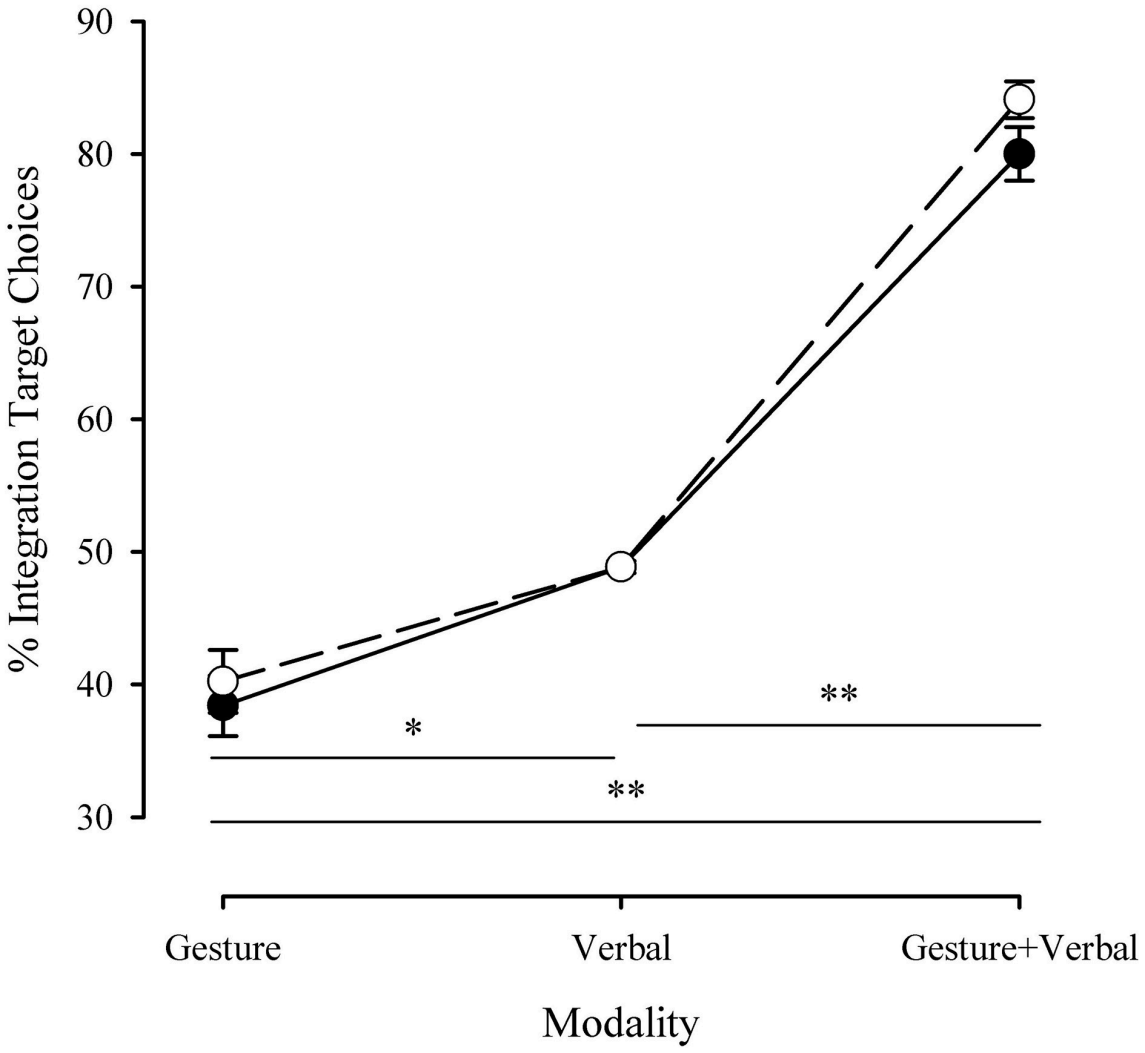
**Proportion of integration target image selection for each communication modality, in dependence of the communication performance medium**. Shaded symbols: robot communication, empty symbols: human communication. Error bars represent ±1 SEM. ^*^p < 0.0005; ^**^p < 0.0001. © 2015 IEEE. Reprinted, with permission, from Bremner and Leonards (2015a).

To test for speech and gesture integration all stimulus item scores were summed for every participant (the scores for a particular phrase were the combined results for the two gestures that accompanied each), hence we determined the proportion of integration target choices (ITC). Figure 8 shows the proportion of participant responses where the integration target was selected, in dependence of the presented stimulus mode.

Uni-modal presentations had a uni-modal image as a correct answer as well as the integration image. In line with expectations that each was equally likely to be chosen, ITC for the verbal condition were made close to 50% of the time; the gesture conditions favored the non-integration target image, with ITC close to 40%. In the multi-modal presentation condition we observed a distinct increase in the frequency with which the integrated image was selected. Underlying the averaged values for uni-modal image selection, a number of individual stimuli had a particular image of the two viable image choices that was chosen significantly more often than the other. In some cases this was the integration target and in some cases it was not; integration target choice in the multi-modal version of those stimuli did not vary significantly from the value found in less extreme uni-modal cases. Hence, this provides stronger evidence for multi-modal integration in cases where a large change occurred. Moreover, this shows the robustness of our approach to these variations as the averaged values are close to those expected.

Accordingly, a 2 (presenter) × 3 (communication modus) repeated measures ANOVA revealed a significant main effect of communication mode [*F*_(2,42)_ = 282.57, *p* < 0.0001]. *Post-hoc* analysis (Tukey) confirmed that participants chose the integrated images far less often in the gesture only condition (*M* = 0.39 ± 0.11*SD*) than in the verbal only condition (*M* = 0.49 ± 0.02*SD, p* < 0.0005). More importantly, participants selected the image constituting the integrated information from speech and gesture in the VG condition (*M* = 0.82 ± 0.08*SD*; *p* < 0.0005). Hence, there there is clear indication that ambiguity is decreased by means of correct integration of speech and gesture information.

We found no significant main effect for presenter [*F*_(1,21)_ = 2.61, *p* = 0.12], nor a significant interaction between communication mode and presenter [*F*_(2,42)_ = 1.23, *p* = 0.30]. This first analysis seems to indicate that integration of information conveyed in speech and gesture is of similar efficiency for a human communication mediated by video or mediated by a robot avatar.

So that we can gain a clearer picture of the pairwise comparisons of integration target image choices, we propose calculation of an estimate of the effect size of changes in ITC proportions in dependence of condition. The method we have utilized to do so is based on the method proposed by Cocks et al. (2011) termed multi-modal gain (*MMG*). *MMG* is a means by which we can estimate the change in probability of ITC between uni-modal (speech or gesture alone) and multi-modal conditions (speech and gesture together). To estimate the value of *MMG*, the proportion of ITC in uni-modal communication (*P*(*Uni*)) is estimated, and then subtracted from the proportion of ITC in the VG conditions (*P*(*Multi*)), see Equation (1).

(1) $$\begin{array}{l} MMG=P\left(Multi\right)-P\left(Uni\right) \end{array}$$

To estimate the proportion of ITC in the uni-modal conditions (*P(Uni)*) the weighted mean of ITC in the verbal (*ITC*_*V*_) and gesture (*ITC*_*G*_) conditions are summed, see Equation (2). The basis for this calculation is that the different modalities vary in how likely they are to be utilized by observers, i.e., it is assumed that participants are more likely to be influenced by the modality that they perceive as providing the most useful information. Thus, the two weights, *WV* and *WG*, for the verbal and gesture conditions respectively, are calculated as normalized proportions of trials in which integration targets were selected (*PCV* for V trials and *PCG* for G trials), see Equations (3) and (4).

(2) $$\begin{array}{r} P\left(Uni\right)=WV*ITC_{V}+WG*ITC_{G} \end{array}$$

(3) $$\begin{array}{r} WV=PCV∕\left(PCV+PCG\right) \end{array}$$

(4) $$\begin{array}{r} WG=PCG∕\left(PCV+PCG\right) \end{array}$$

**Figure 9:**
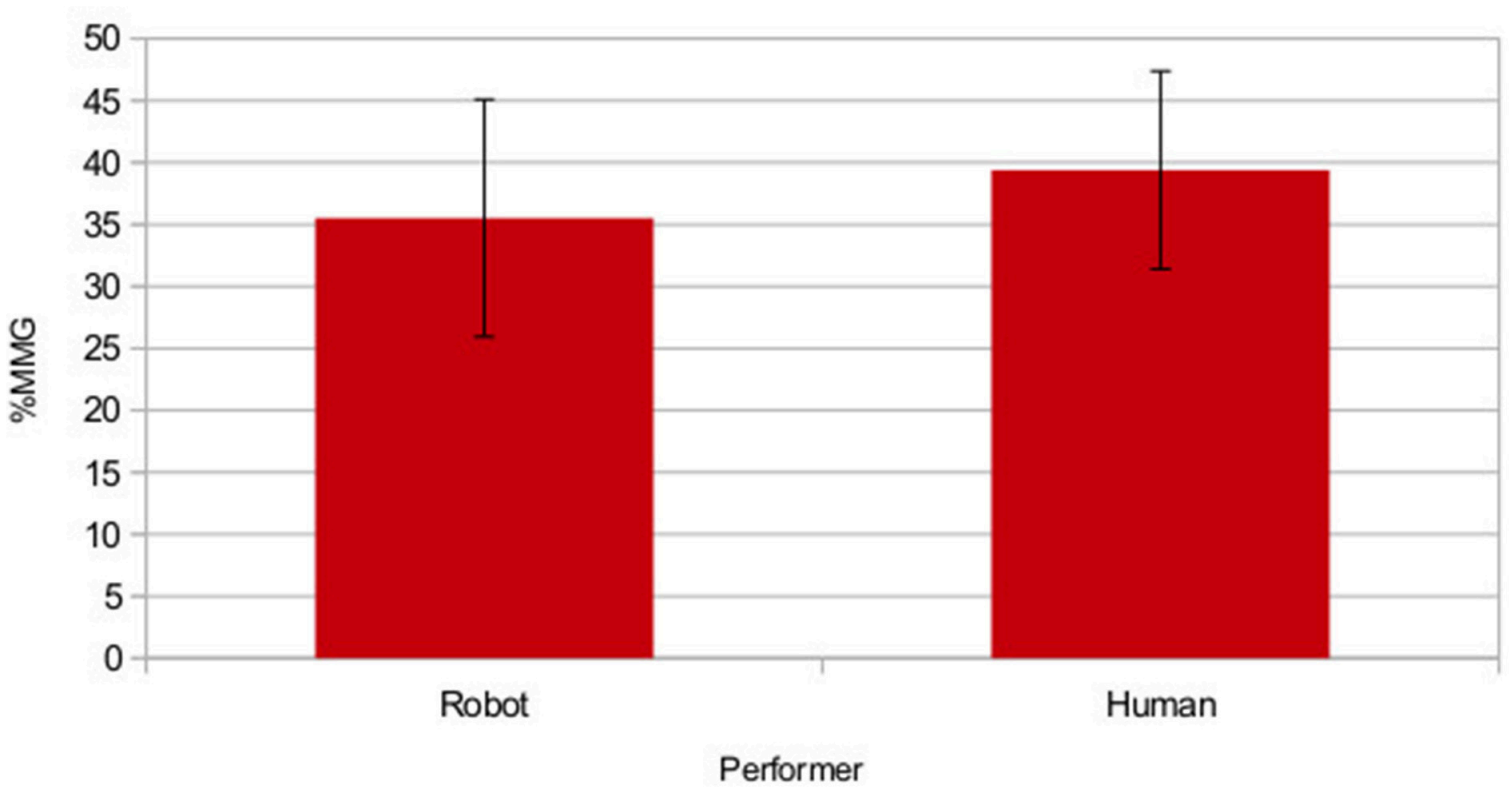
**Group mean multi-modal gain for each performance mode**. Error bars show ±1 SD. © 2015 IEEE. Reprinted, with permission, from Bremner and Leonards (2015a).

Hence, *MMG* calculates a single figure for percentage gain, taking into account how often the integration targets were chosen in both uni-modal conditions (the results for both gestures for each phrase were included together). The values for each performer were calculated separately and are shown in Figure 9. By using two gestures per phrase we found that for some phrases in the verbal condition one of the two matching images was selected far more frequently than the other. Hence, *MMG* for the preferred integration target image was close to zero, i.e., gesture had no effect; conversely, for the other integration target image *MMG* was very high, i.e., gestures had a large effect. This gives us a clear advantage over the original study of Cocks et al. (2011) as we were less vulnerable to the variability of individual meaning preferences, and hence could gain a clearer picture of whether integration effected understanding by incorporating the scores in a single calculation.

We conducted a two tailed *t*-test for each performer against the null hypothesis of *MMG* = 0, the means of both samples (*M*_*H*_ = 0.393 ± 0.079*SD*; *M*_*R*_ = 0.355 ± 0.095*SD*) differed significantly from 0 [*t*_*H*_(21) = 23.12, *p* < 0.001, *r* = 0.98;*t*_*R*_(21) = 17.405, *p* < 0.001, *r* = 0.97]. It is important to be aware that a maximum estimate for *MMG* is given by 1−*P*(*Uni*), hence, *MMG*_*Rmax*_ = 0.56 and *MMG*_*Hmax*_ = 0.56 (i.e., 56 and 55% for the robot and human respectively). The *MMG* values for both performance modes are approaching ceiling.

The means of the two performers were compared using a paired two tailed *t*-test, and this showed no significant differences [*t*_(21)_ = −2.005, *Dif* = 0.019, *p* > 0.05, *r* = 0.21]. However, for testing the hypothesis that there is no difference between performance modes this analysis was underpowered. In order to allow us to more reliably test this hypothesis, i.e., that the performance mode results are interchangeable, a repeatability measure was used, intraclass correlation coefficient (ICC). The *MMG* scores for each participant were calculated from responses in multiple trials (so can be considered akin to a mean score), hence we used *ICC*(2, *k*), as suggested in Shrout and Fleiss (1979). We found significant correlation between the results, indicating fair to substantial reliability [*ICC*(2, *k*) = 0.61, *F*_(21,21)_ = 2.8, *p* = 0.011]. Taking these two analyses together, we thus feel confident that participants' ability to integrate gestures and speech was independent of the performers.

## 4. Discussion

The findings in this paper address the four research questions proposed in Section 1. We found that (1) human observers were able to identify upper limb manner gestures the majority of the time when produced by a tele-operated NAO robot. (2) Although identification of robot-performed gestures was worse than that for human-performed gestures, it was still good enough for them to be useful. More importantly, as gesture in human communication is most commonly employed along with speech, we found that (3) when such gestures were performed with speech they were integrated with it; (4) this process was as efficient for the robot as the human performances. Moreover, this integration compensated for any difficulties in identification of robot performed gestures. In the following sections we will discuss these findings in more detail.

### 4.1. Gesture comprehension

With the exception of those accompanying “I lit,” all gestures used in this experiment were identified clearly above chance for both the human and the robot when they were presented without speech. Though robot gestures were more difficult to identify than human gestures, the general ability to do so is in clear contrast to earlier findings by Cabibihan et al. (2012a) and Zheng and Meng (2012). In both these previous studies they found robot performed gestures were difficult to identify on their own. There are a number of possible causal factors for the differences between our study and previous work. Possible factors are the subtleties in gestures captured by the tele-operation scheme, the different methods of response-gathering (restricted choices as used here, in contrast to free response in related work), the types of gestures used (they used more emblematic gestures, often close to pantomime, in contrast to the iconic manner gestures used here), or some combination of all of these. Whichever the explanatory case, the work presented here provides evidence for the idea that there is communicative value in robot performed gestures.

We suggest that there might be a wider range of gestures than those tested here that will have communicative value for a robot. Therefore, we will look at common features of the gestures used here that were correctly identified. It is also instructive to examine these same features for gestures that were more difficult to identify when performed on the robot than when performed by a human. Differences in the performances likely account for the lower mean recognition rate for robot performed gestures (80.2%, compared to 94.3% for human performances).

The primary common feature is the importance of hand trajectory, including the appropriate hand velocity profile. This is used to convey easily identifiable relative motions that are either part of the action being carried out, or of objects manipulated by the action. This idea is supported by the work of Beattie and Shovelton (2005), who found that gestures portraying relative positions and movements are the most successful at conveying information. Relatedly, when the trajectories could not be correctly perceived gestures were harder to identify. The main reason for this here was due to the reduced range of motion on the NAO elbow flexion, and the lack of the ulnar/radial deviation degree of freedom, resulting in smaller vertical travel for some gestures, and in some cases increased jerk. Moreover, these deviations might also cause difficulties in identifying motion primitives used in gesture recognition (Bengoetxea et al., 2014), or limit the perception of the movement to being artificial where different mental processes are applied (Yang et al., 2015).

One way in which this issue of gesture recognition has been circumvented, is by having participants evaluate gestures not on their meaning alone, but rather on what action they would do in response, as this activates another area of the brain used in gesture recognition (Yang et al., 2015). This was demonstrated in the findings of Riek et al. where in speeded response trials participants were reported to correctly identify responses to robot performed co-operative gestures; they remained able to do so even when the robot used non-human-like velocity profiles (Riek et al., 2010). This suggests that the context in which the gestures are used may be of importance in the ease with which they are recognized.

A second common feature is hand orientation, as different hand orientations for the same hand trajectory can convey very different actions. Indeed, we found that for gestures where the wrist rotation sensor provided erroneous information, those gestures were less frequently correctly identified. As with deviations in arm trajectory this might mean that movement expected according to muscle synergies observed in human gesture (Bengoetxea et al., 2014) is not observed. A final feature, important for robots that do not possess fully articulated hands such as NAO, is a minimal reliance on hand shapes; i.e., gestures where arm trajectories and the degree to which the hand was open or closed contained sufficient information. We found that for some gestures hand shape was required for the gesture not to be too ambiguous to be correctly identified.

A good illustration of the importance of these features can be found in the gesture lit1, which, while being correctly identified in the human presentation condition, was not identified correctly in the robot presentation condition. The lit1 gesture comprises a vertical hand motion demonstrating pulling a cord to switch on a light (a common action in the UK). In the robot condition the unrelated foil images were selected with close to identical frequency as the target images. Examining the response image set for “I lit,” we observed that the main differences between target and foil images was hand orientation, and motion range. Hence, we suggest, if gesture is to be used in uni-modal communication for a robot, as an avatar or autonomously, which gestures are used needs to be carefully examined, and the capabilities of the robot platform taken into account.

While the evidence for the relevance of the aforementioned deviations is limited, it does highlight an important factor both for gestures in HRI and in human communication that merit further investigation. We suggest this key factor is that the differences between human and robot gestures are relatively small, as shown in the performance analysis of the tele-operation control scheme in producing closely matched joint motion. Hence, our data provide further evidence for the notion that people are well conditioned to making subtle gestural discriminations and to identify biological motion and meaning (Kilner et al., 2003; Yang et al., 2015). This is further reinforced by our observations during the development of the range of gestures to be tested.

To test how susceptible observers are to subtle variations in robot performed gesture and how much this depends on the context (e.g., whether observers are needed to physically or socially interact with the robot) requires more compelling evidence (see also Riek et al., 2010). Further, whether such effects vary between deliberate gesture identification, and the use of such gestures in conversation, also needs to be investigated. Indeed, by testing subtle gesture effects for robot communication we may be able to also learn more about the mechanisms underlying human communication and gesture perception.

### 4.2. Speech and gesture integration

Our findings demonstrate that when performed together speech and gesture are integrated, even when performance is mediated by a tele-operated NAO robot. We observed a larger proportion of integration target choices (ITC) in the multi-modal condition, as compared to either uni-modal condition. Multi-modal communication disambiguates the possible meaning of either gesture or speech on their own. ITC differed between uni-modal conditions, making it difficult to directly evaluate and compare the extent of speech and gesture integration for the two performers. To overcome this difficulty we followed the methodology of Cocks et al. to calculate multi-modal gain (*MMG*) (Cocks et al., 2011). *MMG* incorporates the results from both uni-modal presentation conditions in a calculation to estimate the change in probability of ITC for multi-modal communication as a single value. Highly significant values for *MMG* were found for both performance conditions. More importantly, the extent to which speech and gesture could be integrated was comparable between the two performers, indicating that robot-performed gestures are as efficiently integrated with speech as human-performed multi-modal communication.

As the lit gestures were not identified correctly when presented alone by the robot, it is instructive to examine the image choices when presented alongside speech. For lit1 and lit2 gestures, the correct target image was selected by 82% of participants and 95% of participants, respectively. This shows that participants were able to compensate for the lack of clarity in the gesture performance by using speech information to resolve ambiguity.

These results are somewhat surprising given previous work on speech and gesture integration with mismatched appearance and voice (here there is a clear mismatch of human voice and robot appearance). Kelly et al. showed that when there was a gender mismatch between voice and gesture performer, integration was reduced, and required considered rather than automatic mental processing (Kelly et al., 2010). Hayes et al. replicated these findings with human voice and robot performed gestures (Hayes et al., 2013). Similarly, we found that in speeded trials integration of speech and beat gestures does not occur when using a robot avatar to communicate (Bremner and Leonards, 2015b). The work presented here differs from the aforementioned, in that trials were not speeded.

We suggest that though integration of robot gesture and human speech may not be an automatic process, it occurs nevertheless. Whether there is a difference in mental processing for the gestures examined here, and if there is, whether it effects interaction with robot tele-operators requires further investigation. One way in which this could be tested is to look not only at information comprehension, but also response times in speeded trials.

As well as being important for tele-communication using humanoid robot avatars, our findings also have implications for design of communicative behavior in autonomous humanoid robots. Perhaps the most important implication is that when a humanoid robot needs to communicate this can be done more accurately and efficiently by splitting semantic information across verbal and gestural communication modalities. In addition, our results demonstrate that multi-modal communications are interpreted similarly whether the gestural component is mediated by video only or by a tele-operated robot. Hence, autonomous robots should, where possible, use gestures to produce more natural seeming human-robot interaction. Thus, our work reinforces findings in the literature that higher subjective ratings are given to robots when they perform gestures (Han et al., 2012; Aly and Tapus, 2013; Salem et al., 2013).

Importantly, the difference in gesture recognition between human video and robot-embodied communication for gesture only communication is compensated for in multi-modal communication. That is to say, a humanoid robot avatar offers comparable performance to video communication when using speech along with gestures. Hence, a robot avatar operator might take advantage of previously observed advantages of robots over 2D communication media, such as enhanced engagement, improved social presence and action awareness (Powers et al., 2007; Adalgeirsson and Breazeal, 2010; Hossen Mamode et al., 2013), while maintaining communicative efficacy.

### 4.3. Conclusion

We show in this paper, using a fully within subject design, that using our Kinect based tele-operation system iconic manner gestures conveyed on the NAO robot are recognizable. This is despite physical restrictions in the degrees of freedom and movement kinematics of NAO relative to a human. Further, there seem to exist a large range of gestures which might be conveyed successfully. More importantly, we show that such robot-executed gestures can be integrated with simultaneously presented speech as efficiently as human-executed gestures. Whether this is because of, or despite the speech clearly originating from a human operator, remains to be further investigated. Hence, with regard to multi-modal semantic information conveyance, a NAO tele-operated avatar can be close to video mediated human communication in terms of efficacy. These two findings provide strong evidence as to the utility of a tele-operated NAO for conveying multi-modal communication. Although gestures are not recognized quite as well for the robot as they are for the human on video, they are still recognized well enough to make it a viable communication medium. We suggest the slight compromise in uni-modal gesture recognition for a robot performer is compensated for by the potential improvements in social presence and salience to interlocutors.

Our findings also have implications for autonomous communication robots, for which gesturing is an active area of research, and has been shown to offer a number of communicative benefits beyond information conveyance. Huang and Mutlu found that robot performed deictic gestures improved participants' recall of items in a factual talk; however, gestures other types had minimal effects (Huang and Mutlu, 2014). Bremner et al. showed that although higher certainty in the information recalled was observed for parts of a monolog that were accompanied by (beat and metaphoric) gestures, the amount of information recalled was no better than for parts without gesture (Bremner et al., 2011). However, Van Dijk et al. found there was a positive influence on memory when redundant iconic gestures were performed when describing action performance (Dijk et al., 2013).

Other gesture effects beyond memory have been observed by Chidambaram et al. (2012), who demonstrated a robot was significantly more persuasive when it used gestures and other non-verbal cues. Additionally, hand gestures have been found to improve user ratings of robots on scales such as competence, likeability, and intention for future contact in a number of studies (e.g., Han et al., 2012; Aly and Tapus, 2013; Salem et al., 2013). These findings suggest that performing gestures on a robot avatar may have additional benefits to the robot operator that can be capitalized on, and we are in a position to do so now that we have shown they can be interpreted correctly.

We suggest that, when it is possible, robot communication should be multi-modal to ensure clarity of meaning, and to improve its efficiency and efficacy. This demonstration of the utility of multi-modal communication is not only of importance for our continuing work with tele-operated humanoid robot avatars, but also for socially communicative autonomous humanoid robots. We suggest our results might be generalizable in this way as previous studies showed that participants treat avatars similarly to how they do autonomous systems (von der Pütten et al., 2010). Indeed, one of the applications of humanoid tele-operation is as a tool to test what is important in terms of robot behavior for successful HRI in so-called super Wizard of Oz studies (Gibert et al., 2013).

### 4.4. Limitations and future work

While the work presented here provides initial insight into speech and iconic gesture integration for robotic communicators, it has a number of limitations which we hope to address in future work. Firstly, the range of tested gestures was limited to manner gestures where hand shape was not expected to be critical. In the future we intend to expand on our findings that integration can occur even for gestures that, as a consequence of differences in physical capabilities, can not be realized in a precisely human-like way by a robot. Limited evidence was found for this with the “I lit” gestures which were poorly recognized when performed by the robot.

The degree of similarity between robot performed and the original human gestures was not objectively controlled, other than visual inspection. Given our preliminary findings on the effects of subtle gesture differences, and existing literature on human sensitivity to biological motion, we suggest the examination of the degree of similarity required for comprehension and integration. Doing so would inform robot design and control requirements (extending the ideas in Riek et al., 2010). Additionally, we suggest that by both carefully controlling gesture motion requirements, and similarity to human motion, one could more easily generalize our results across different robot platforms.

Another limitation of our work was that all gestures used were tested in a laboratory setting, with a limited set of short communications. In future work we aim to improve the ecological validity of our findings by investigating gestures in more interactive settings (extending the ideas in Hossen Mamode et al., 2013). In doing so we aim to look at a larger range of types of gesture, situated within longer sentences, and accompanied by other non-verbal behaviors such as gaze. An important component of this further work will be timing of gestures relative to speech (McNeill, 1992; Kendon, 2004). Though initial testing has shown coordination between speech and gesture to be close to that of the robot operator, whether it is close enough needs to be experimentally verified to fully validate our robot avatar system as a communication medium.

It is also important to note that our results might not be generalizable across cultures. Different nationalities have different gesturing conventions, and semantics (i.e., words that are ambiguous in English are often not in other languages). Further work is required to see if integration varies across different cultures, particularly where gestures are more (e.g., Italy), or less (e.g., Japan) prevalent in everyday communication.

## Author contributions

PB, conception and design of the work; acquisition, analysis, and interpretation of data for the work; drafting of the manuscript. UL, conception and design of the work; analysis, and interpretation of data for the work; revising work critically for important intellectual content. PB and UL, final approval and accountability.

## Funding

This research grant is funded by the EPSRC under its IDEAS Factory Sandpits call on Digital Personhood, grant ref: EP/L00416X/1.

### Conflict of interest statement

The authors declare that the research was conducted in the absence of any commercial or financial relationships that could be construed as a potential conflict of interest.

## Footnotes

^1^calculations are omitted here for brevity as they are relatively trivial.

^2^For access to results data pertaining to this work please contact the lead author.

## References

[B1] Adalgeirsson S. O. Breazeal C. (2010). MeBot: a robotic platform for socially embodied telepresence, in Proceedings of International Conference Human Robot Interaction (Osaka: ACM/IEEE), 15–22. 10.1109/hri.2010.5453272

[B2] Aly A. Tapus A. (2013). A model for synthesizing a combined verbal and nonverbal behavior based on personality traits in human-robot interaction, in Proceedings of International Conference Human Robot Interaction (Tokyo: ACM/IEEE), 325–332. 10.1109/hri.2013.6483606

[B3] Baillie J.-C. Demaille A. Hocquet Q. Nottale M. Tardieu S. (2008). The urbi universal platform for robotics, in First International Workshop on Standards and Common Platform for Robotics (Venice).

[B4] Beattie G. Shovelton H. (2005). Why the spontaneous images created by the hands during talk can help make TV advertisements more effective. Br. J. Psychol. 96, 21–37. 10.1348/000712605X103500 15826322

[B5] Beattie G. Shovelton H. (2011). An exploration of the other side of semantic communication: how the spontaneous movements of the human hand add crucial meaning to narrative. Semiotica 184, 33–51. 10.1515/semi.2011.021

[B6] Bengoetxea A. Leurs F. Hoellinger T. Cebolla A. M. Dan B. Cheron G.. (2014). Physiological modules for generating discrete and rhythmic movements: component analysis of EMG signals. Front. Comput. Neurosci. 8:169. 10.3389/fncom.2014.00100 25620928 PMC4288127

[B7] Bremner P. Leonards U. (2015a). Efficiency of speech and iconic gesture integration for robotic and human communicators—a direct comparison, in Proceedings of IEEE International Conference on Robotics and Automation (Seattle, WA: IEEE), 1999–2006. 10.1109/icra.2015.7139460

[B8] Bremner P. Leonards U. (2015b). Speech and gesture emphasis effects for robotic and human communicators, in Proceedings of the Tenth Annual ACM/IEEE International Conference on Human-Robot Interaction (Portland, OR: ACM Press), 255–262. 10.1145/2696454.2696496

[B9] Bremner P. Pipe A. G. Melhuish C. Fraser M. Subramanian S. (2011). The effects of robot-performed co-verbal gesture on listener behaviour, in 11th IEEE-RAS International Conference on Humanoid Robots, (IEEE), 458–465. 10.1109/humanoids.2011.6100810

[B10] Cabibihan J.-J. So W.-C. Pramanik S. (2012a). Human-recognizable robotic gestures. IEEE Trans. Autonom. Mental Dev. 4, 305–314. 10.1109/TAMD.2012.2208962

[B11] Cabibihan J.-J. So W.-C. Saj S. Zhang Z. (2012b). Telerobotic pointing gestures shape human spatial cognition. Int. J. Soc. Robot. 4, 263–272. 10.1007/s12369-012-0148-9

[B12] Cassell J. McNeill D. McCullough K.-E. (1999). Speech-gesture mismatches: evidence for one underlying representation of linguistic and nonlinguistic information. Pragmat. Cogn. 7, 1–34. 10.1075/pc.7.1.03cas

[B13] Chidambaram V. Chiang Y.-H. Mutlu B. (2012). Designing persuasive robots: how robots might persuade people using vocal and nonverbal cues, in Human-Robot Interaction (HRI), 2012 7th ACM/IEEE International Conference on, (IEEE), 293–300. 10.1145/2157689.2157798

[B14] Cocks N. Morgan G. Kita S. (2011). Iconic gesture and speech integration in younger and older adults. Gesture 11, 24–39. 10.1075/gest.11.1.02coc

[B15] Dijk E. T. Torta E. Cuijpers R. H. (2013). Effects of eye contact and iconic gestures on message retention in human-robot interaction. Int. J. Soc. Robot. 5, 491–501. 10.1007/s12369-013-0214-y

[B16] Ekman P. (1976). Movements with precise meanings. J. Commun. 26, 14–26. 10.1111/j.1460-2466.1976.tb01898.x

[B17] Gazzola V. Rizzolatti G. Wicker B. Keysers C. (2007). The anthropomorphic brain: the mirror neuron system responds to human and robotic actions. NeuroImage 35, 1674–1684. 10.1016/j.neuroimage.2007.02.003 17395490

[B18] Gibert G. Petit M. Lance F. Pointeau G. Dominey P. F. (2013). What makes humans so different? Analysis of human-humanoid robot interaction with a super wizard of oz platform, in Towards Social Humanoid Robots: What makes Interaction Human-Like? Workshop at International Conference on Intelligent Robots and Systems (Tokyo).

[B19] Gouaillier D. Hugel V. Blazevic P. Kilner C. Monceaux J. Lafourcade P.. (2009). Mechatronic design of NAO humanoid, in Proceedings of IEEE International Conference on Robotics and Automation (Kobe: IEEE), 769–774. 10.1109/robot.2009.5152516

[B20] Han J. Campbell N. Jokinen K. Wilcock G. (2012). Investigating the use of non-verbal cues in human-robot interaction with a Nao robot, in 2012 IEEE 3rd International Conference on Cognitive Infocommunications (CogInfoCom) (Košice: IEEE), 679–683. 10.1109/CogInfoCom.2012.6421937

[B21] Hayes C. J. Crowell C. R. Riek L. D. (2013). Automatic processing of irrelevant co-speech gestures with human but not robot actors, in Proceedings of the 8th ACM/IEEE International Conference on Human-Robot Interaction (Tokyo: IEEE Press), 333–340. 10.1109/HRI.2013.6483607

[B22] Hossen Mamode H. Z. Bremner P. Pipe A. G. Carse B. (2013). Cooperative tabletop working for humans and humanoid robots: group interaction with an avatar, in IEEE International Conference on Robotics and Automation (Karlsruhe: IEEE), 184–190. 10.1109/icra.2013.6630574

[B23] Hostetter A. B. (2011). When do gestures communicate? a meta-analysis. Psychol. Bull. 137, 297–315. 10.1037/a0022128 21355631

[B24] Huang C.-M. Mutlu B. (2014). Learning-based modeling of multimodal behaviors for humanlike robots, in Proceedings of the 2014 ACM/IEEE International Conference on Human-Robot Interaction-HRI'14, (Bielefeld: ACM Press), 57–64. 10.1145/2559636.2559668

[B25] Kelly S. D. Barr D. J. Church R. Lynch K. (1999). Offering a hand to pragmatic understanding: the role of speech and gesture in comprehension and memory, J. Mem. Lang. 40, 577–592. 10.1006/jmla.1999.2634

[B26] Kelly S. D. Creigh P. Bartolotti J. (2010). Integrating speech and iconic gestures in a Stroop-like task: evidence for automatic processing. J. Cogn. Neurosci. 22, 683–694. 10.1162/jocn.2009.21254 19413483

[B27] Kendon A. (2004). Gesture: Visible Action as Utterance. Cambridge, UK: Cambridge University Press.

[B28] Kilner J. M. Paulignan Y. Blakemore S. J. (2003). An interference effect of observed biological movement on action. Curr. Biol. 13, 522–525. 10.1016/S0960-9822(03)00165-9 12646137

[B29] McNeill D. (1992). Hand and Mind: What Gestures Reveal about Thought. Chicago, IL: University of Chicago Press.

[B30] Metta G. Fitzpatrick P. Natale L. (2006). Yarp: yet another robot platform. Int. J. Adv. Robot. Syst. 3, 43–48. 10.5772/5761

[B31] Ono T. Kanda T. Imai M. Ishiguro H. (2003). Embodied communications between humans and robots emerging from entrained gestures, in Proceedings 2003 IEEE International Symposium on Computational Intelligence in Robotics and Automation (Kobe: IEEE), 558–563. 10.1109/CIRA.2003.1222241

[B32] Peirce J. W. (2007). PsychoPy–Psychophysics software in Python. J. Neurosci. Methods 162, 8–13. 10.1016/j.jneumeth.2006.11.017 17254636 PMC2018741

[B33] Powers A. Kiesler S. Fussell S. Torrey C. (2007). Comparing a computer agent with a humanoid robot, in Human-Robot Interaction (HRI), 2007 2nd ACM/IEEE International Conference on, (IEEE), 145–152. 10.1145/1228716.1228736

[B34] Quigley M. Conley K. Gerkey B. Faust J. Foote T. Leibs J.. (2009). {ROS}: an open-source Robot Operating System, in Open-Source Software Workshop of the International Conference on Robotics and Automation (ICRA) (Shanghai).

[B35] Riek L. Rabinowitch T. Bremner P. Pipe A. Fraser M. Robinson P. (2010). Cooperative gestures: effective signaling for humanoid robots, in 5th ACM/IEEE International Conference on Human-Robot Interaction (Osaka). 10.1145/1734454.1734474

[B36] Salem M. Eyssel F. Rohlfing K. Kopp S. Joublin F. (2013). To Err is human(-like): effects of robot gesture on perceived anthropomorphism and likability. Int. J. Soc. Robot. 5, 313–323. 10.1007/s12369-013-0196-9

[B37] Sauppé A. Mutlu B. (2014). Robot deictics, in Proceedings of the 2014 ACM/IEEE International Conference on Human-Robot Interaction-HRI'14 (Bielefeld: ACM Press), 342–349. 10.1145/2559636.2559657

[B38] Shrout P. E. Fleiss J. L. (1979). Intraclass correlations: uses in assessing rater reliability. Psychol. Bull. 86, 420–428. 10.1037/0033-2909.86.2.420 18839484

[B39] Tanaka K. Nakanishi H. Ishiguro H. (2015). Physical embodiment can produce robot operator's pseudo presence. Front. ICT 2:8 10.3389/fict.2015.00008

[B40] von der Pütten A. M. Krämer N. C. Gratch J. Kang S.-H. (2010). It doesn't matter what you are! Explaining social effects of agents and avatars, Comput. Hum. Behav. 26, 1641–1650. 10.1016/j.chb.2010.06.012

[B41] Wang L. Chu M. (2013). The role of beat gesture and pitch accent in semantic processing: an ERP study. Neuropsychologia 51, 2847–2855. 10.1016/j.neuropsychologia.2013.09.027 24060845

[B42] Yang J. Andric M. Matthew M. M. (2015). The neural basis of hand gesture comprehension: a meta-analysis of functional magnetic resonance imaging studies. Neurosci. Biobehav. Rev. 57, 88–104. 10.1016/j.neubiorev.2015.08.006 26271719

[B43] Zheng M. Meng M. Q.-H. (2012). Designing gestures with semantic meanings for humanoid robot, in 2012 IEEE International Conference on Robotics and Biomimetics (ROBIO) (Guangzhou: IEEE), 287–292. 10.1109/ROBIO

